# Biomarkers for Predicting Efficacies of Anti-PD1 Antibodies

**DOI:** 10.3389/fmed.2019.00174

**Published:** 2019-07-31

**Authors:** Yumi Kambayashi, Taku Fujimura, Takanori Hidaka, Setsuya Aiba

**Affiliations:** Department of Dermatology, Tohoku University Graduate School of Medicine, Sendai, Japan

**Keywords:** anti-PD1 antibodies, routine blood test, LDH, MSH, TMB, TAM-related factors

## Abstract

Therapeutic options for treating advanced melanoma are progressing rapidly. Although anti-programmed cell death 1 (PD1) antibodies (e.g., nivolumab, pembrolizumab) have been approved as first-line and anchor drugs, respectively, for treating advanced melanoma, the efficacy appears limited as we expected, especially in Asian populations. Biomarkers to predict or evaluate the efficacy of anti-PD1 antibodies are needed to avoid subjecting patients to potentially severe adverse events associated with switching to other anti-melanoma drugs. This review focuses on the recent development of biomarkers for assessing the efficacy of anti-PD1 antibodies using routine blood tests such as the neutrophil-to-lymphocyte ratio, eosinophil ratio, serum markers such as lactate dehydrogenase, programmed cell death ligand 1 (PD-L1) expression on melanoma cells, microsatellite instability and mismatch repair deficiency assays, as well as soluble CD163, and tumor-associated macrophage-related chemokines (e.g., CXCL5, CXCL10).

## Introduction

Anti-programmed cell death 1 (PD-1) antibodies are in wide use for the treatment of various cancers, particularly cancers with a high tumor mutation burden (TMB) such as advanced cutaneous melanoma (1–4). Although BRAF inhibitors in combination with MEK inhibitors are useful for the treatment of BRAF^V600^-mutant advanced melanoma, the population of BRAF^V600^-mutant advanced melanoma is limited, particularly in the Japanese population, which contains large populations with acral lentiginous melanoma and mucosal melanoma (5, 6). Most patients with advanced melanoma are therefore administered nivolumab with or without ipilimumab, or pembrolizumab as a first-line therapy.

Ipilimumab is a fully humanized immunoglobulin (Ig)G1 monoclonal antibody that blocks cytotoxic T-lymphocyte antigen (CTLA-4) to activate and increase T cells, particularly the tumor-recognized T-cell clones that reside in primary tumors (7, 8). Combination therapy comprising nivolumab and ipilimumab or sequential administration of nivolumab and ipilimumab with a planned switch are among the most effective chemotherapies against advanced melanoma (9–11), and even increase the response rate (RR) for untreated metastasis of melanoma to the brain compared to nivolumab monotherapy (12). On the other hand, the efficacy of ipilimumab in patients with nivolumab-resistant melanoma is low after objective tumor progression compared to planned-switched patients (13). In addition, ipilimumab leads to a high frequency of immune-related adverse events (irAEs) among patients with advanced melanoma, particularly combination therapy with nivolumab (9, 11). Taken together, evaluation of the efficacy of these treatments in advance is important.

This review focuses on the recent development of biomarkers for assessing the efficacy of anti-PD1 antibodies using routine blood tests such as the neutrophil-to-lymphocyte ratio, eosinophil ratio, serum markers such as lactate dehydrogenase (LDH), PD-L1 expression on melanoma cells, microsatellite instability (MSI) and mismatch repair deficiency assays, as well as soluble CD163, and tumor-associated macrophage (TAM)-related chemokines (e.g., CXCL5, CXCL10) (Table 1).

**Table 1:** Highlighted papers in each chapter.

	**Interest**	**Considerable interest**
Routine blood test	14, 21, 25	23
PD-L1 expression	31	32
MSI and TMB	40, 43	3, 39
TAMs related factors	24, 50	48

## Significance of Routine Blood Tests for Predicting the Efficacy of Anti-Pd1 Antibody

### Leukocyte-to-Lymphocyte Ratio (LLR), Neutrophil-to-Lymphocyte Ratio (NLR), Monocyte Count, and Absolute Lymphocyte Count (ALC)

Recent reports have suggested the significance of routine blood tests, such as cell counts and cell ratios, for predicting the efficacy of anti-PD1 antibodies against advanced melanomas (14–18). Indeed, Fujisawa et al. reported that increased baseline NLR combined with serum LDH was significantly correlated with the efficacy rate of nivolumab according to multivariate analysis, and negatively correlated with efficacy of nivolumab for advanced melanoma (14). In another report, Chasseuil et al. found that increased monocyte count was significantly associated with decreased overall survival (OS) and progression-free survival (PFS) in patients with advanced melanoma according to multivariate analysis. In addition, they also reported that LLR was significantly associated with decreased OS (15). In addition, Rosner et al. reported that not only a low NLR, but also high proportion of eosinophils, high proportion of basophils, low absolute monocyte count and low LDH might be independently associated with favorable OS (16). Since several previous reports have also suggested that NLR is significantly correlated with the efficacy of ipilimumab in the treatment of melanoma patients (17, 18), baseline NLR could be one possible predictive marker for immune checkpoint inhibitor (ICI)-treated patients with advanced melanoma.

Lower ALC shows significantly less clinical benefit from anti-PD1 antibody (19), which is associated with pretreatment NLR in patients with head and neck squamous cell carcinoma. They concluded that patients with pretreatment ALC <600 cells/μl had shorter PFS than patients with pretreatment ALC ≥600 cells/μl. In another report, Soyano et al. retrospectively analyzed 157 patients with advanced non-small cell lung cancer (NSCLC) treated with anti-PD1 antibodies using logistic regression analysis, suggesting that a high baseline NLR correlated significantly with increased risks of death and disease progression (20). In addition, they also reported that a high baseline myeloid-to-lymphoid cell ratio significantly increased the risk of death, even after multivariate analysis [hazard ratio (HR) = 2.31, *p* = 0.002]. Indeed, a meta-analysis of 14 retrospective studies that had examined the benefits of nivolumab in patients with NSCLC suggested an association of high NLR with poor PFS and OS after nivolumab treatment (21). Moreover, they also reported that post-treatment NLR acted as a predictor of PFS and OS. Overall, these reports have suggested that baseline routine blood tests are important for predicting the efficacy of ICI (Table 2).

**Table 2:** Summary of biomarkers and their efficacy.

**Treatment**	**Patients number**	**Subpopulation**	**Outcome**	**Marker**	**Result (95% Cl) RR = 14%**	***p*-value**	**References**
Nivolumab	*n =* 90	LDH (upper normal limit)	RR	NLR > 2.2 NLR < 2.2	RR = 14% RR = 57%	<0.05	(14)
		LDH normal	RR	NLR > 2.2 NLR < 2.2	RR = 37% RR = 67%	<0.001	
Nivolumab	*n =* 87		OS	Monocyte count (upper normal limit)	HR = 4.31 (1.46–12.74)	0.01	(15)
			PFS	Monocyte count (upper normal limit)	HR = 3.5 (1.01–12.1)	0.04	
Nivolumab + lpllimumab	*n =* 209		OS	NLR Eosinophils Basophils Absolute monocyte LDH (246 <)	HR = 1.95 (111–3.47) HR = 2.38 (1.27–4.46) HR = 1.86 (0.94–3.66) HR = 2.75 (1.30–5.80) HR = 3.71(2.08–6.61)	0.02 0.007 0.08 0.01 <0.0001	(16)
Lpllimumab	*n =* 183		OS	Baseline NLR NLR (end of treatement)	HR = 1.06 (1.01–1.10) HR = 1.06 (1.02–1.09)	0.016 <0.001	(17)
Lpllimumab	*n =* 720		OS PFS	ANC ANC	HR = 3.38 (2.62–4.36) HR = 2.52 (1.97–3.21)	<0.0001 <0.0001	(18)
Anti-PD1 antibody	*n =* 66		OS	LDH elevated LDH normal	4.3 months 15,7 months	<0.00623	(22)
Nivolumab	*n =* 210		RR	PD-L1 positive PD-L1 negative	52.7% (40.8–64.3) 33.1% (25.2–41.7)		(23)
Nivolumab	*n =* 59		RR	Increased Scd163	Sensitivity 84.6% Specificity 87.0%	<0.0030	(24)

*ANC, absolute neutrophilcount; HR, hazzard ratio; NLR, neutrophilto lymphocyte ratio; OS, overall survival; PFS, progress free survival; RR, response rate; UNL, under normal limit*.

### Clinical Use of LDH

Generally, large baseline tumor size in parallel with increased levels of LDH correlates with poor prognosis in advanced melanoma patients (25). Diem et al. first reported the benefit of measuring serum LDH in 66 patients with advanced melanoma treated using anti-PD1 antibody (22). Indeed, patients with elevated baseline LDH showed significantly shorter OS compared to patients with normal LDH. Moreover, they suggested serum LDH as a useful marker during treatment for predicting both early response of anti-PD1 antibody and progressive disease (22).

ECOG performance status (PS) and elevated LDH were reported as independent variables significantly associated with poor OS (26). More recently, Wagner et al. reported serum LDH levels and S100B among the early prognostic markers for response and OS in advanced melanoma patients treated with ICI (27). They concluded that, compared with patients showing normal LDH, increased serum LDH (>25%) was significantly associated with impaired OS when co-existing with increased serum levels of S100B (27). Increased LDH correlated with the poor prognostic factors of not only cutaneous melanoma, but also uveal melanoma, which possesses a high potential for rapid metastasis (28), and NSCLC (29). Since anti-PD1 antibody applies to various cancers, including gastric cancer, renal cell carcinoma, and Hodgkin lymphoma, measurement of LDH might offer a useful, standard marker for patients treated using ICI.

## Expression Levels of PD-L1

In cutaneous melanoma, both tumor cells and TAMs express PD-L1, leading to the maintenance of an immunosuppressive microenvironment at tumor sites (30, 31). Hino et al. first reported PD-L1 expression on melanoma cells as an independent prognostic factor that correlates with vertical invasion of melanoma cells (31). Accordingly, many studies have suggested that PD-L1 expression on melanoma cells can represent a biomarker for predicting the efficacy of anti-PD1 antibodies (32, 33), and even other ICIs (34). For example, PD-L1 expression on melanoma cells in pretreatment tumor biopsy samples correlated with RR, PFS, and OS in advanced melanoma patients treated using anti-PD1 antibodies (33). In another report, expression of PD-L1 correlated with 24-month survival rate in patients with advanced melanoma treated with pembrolizumab (32). Indeed, median PFS in patients with PD-L1 positive melanoma cells was 6.6 months (95% confidence interval (CI), 4.2–9.7 months), while median PFS in patients with PD-L1-negative melanoma cells was 2.8 months (95%CI, 2.8-3.7 months) (32). On the other hand, Hodi et al. reported that assessment of the expression of PD-L1 alone offers a poor predictor of OS in patients treated with nivolumab or nivolumab in combination with ipilimumab (CheckMate 067) (34). Notably, even in PD-L1-negative or -intermediate expressing groups, the RR is still high (33.1%; 95%CI, 25.2-41.7%), suggesting that PD-L1 expression might represent an independent prognostic factor (35). Although those reports suggested the clinical benefits of assessing PD-L1 expression on melanoma cells in predicting the clinical outcomes of ICI treatment, the clinical utility in the real world is limited because of the low sensitivity of immunohistochemical (IHC) assays using different antibody clones, staining platforms and scoring systems in each institute (32–36). To avoid misprediction by IHC staining, more recently, Conroy et al. tried to assess the expression of PD-L1 using next-generation RNA sequencing, but the sensitivity of their system resembles that of IHC assay systems (36). In future, additional assays will be needed to improve the sensitivity of PD-L1 analysis in the prediction of clinical outcomes for ICI treatment of melanoma.

## MSI and TMB

The high RR to anti-PD1 antibodies for cancers with high frequency of MSI has been highlighted in many recent clinical studies (37, 38). Among cancer species, colorectal cancer and endometrial cancer possess a high frequency of MSI (approx. 20~33%) (38, 39), leading to the results of clinical studies that have presented significantly improved RR, PFS and OS in patients with mismatch-repair deficient colorectal cancers compared to those of mismatch repair-proficient colorectal cancers (37–39). Recent reports have also suggested that high infiltration of T-helper 1 (Th1) cells and cytotoxic T lymphocytes (CTLs) produce substantial amounts of interferon gamma (IFNγ), leading to increased expression of PD-L1 in tumors with a high frequency of MSI (37, 40). As described above, since high expression of PD-L1 can provide a biomarker for predicting the efficacy of anti-PD1 antibodies, a high frequency of MSI could correlate with RR, PFS and OS following use of anti-PD1 antibodies.

High TMB correlated with increased neoantigens in various cancers, and could provide predictors for the efficacy of ICI treatment (1, 2, 41). For example, cutaneous squamous cell carcinoma (cSCC) possesses a high TMB (50 mutations per megabase DNA pairs) (42), leading to a high RR for cemiplimab [47% (95%CI, 34–61%)] (43). In addition, since an ultraviolet (UV) damage subclass of SCC and sebaceous carcinoma harbors a high somatic mutation burden with >50 mutations per megabase, UV damage signatures in TMB in these skin cancers could be predictive biomarkers for ICI treatment (44, 45). In melanoma, Madore et al. reported that a lower non-synonymous mutation burden correlated with negative results for PD-L1 expression on melanoma cells, and significantly worse melanoma-specific survival in stage III melanoma (HR = 0.28; 95%CI, 0.12-0.66; P = 0.002) (3). In addition, significant increases in the gene expression signatures of cytotoxic T-cell (CTL) and macrophage-specific genes were seen in PD-L1-positive melanomas, correlating with better melanoma-specific survival (HR = 0.2; 95%CI, 0.05-0.87; *P* = 0.017). Taken together, those reports might suggest the significance of assessing TMB before the administration of ICIs, especially anti-PD1 antibodies, although further studies are needed to confirm its effectiveness.

## Pilot Study for Predictable Biomarkers: TAM-Related Factors (sCD163, CXCL5)

TAMs are functionally reprogrammed to polarized phenotypes by exposure to various factors, leading to the maintenance of a tumor microenvironment (30). Expression of PD-L1 on TAMs is modified by both stromal factors such as regulatory T cells (Tregs) and exogenous factors including immune therapies (46). For example, in a mouse melanoma model (ret, B16 melanoma), depletion of Tregs decreased PD-L1, B7H3, and B7H4 expression on TAMs *in vivo* (46). In patients with esophageal carcinoma, a high density of CD163+ TAMs, which is also associated with significantly increased PD-L1 expression (47), was associated with significantly worse OS than a low density (log-rank *P* = 0.0025) (47). That report suggested that a high density of PD-L1-expressing CD163+ TAMs could offer a prognostic biomarker for esophageal carcinoma (47). Since PD-L1 on tumor cells could be one prognostic factor for melanoma patients treated with ICIs (as described in Chapter 3), TAM-related factors could offer biomarkers for predicting the efficacy of ICI.

TAMs in melanoma patients express not only PD-L1, but also PD-1 (48). Because PD-1 expression in TAMs is one of the key factors in M2 macrophage polarization (49), administration of an anti-PD1 antibody might repolarize TAMs, leading to TAM activation in melanoma patients. Notably, the main population of TAMs in skin cancer is CD163+ M2 macrophages, with soluble (s)CD163 as the activation marker (14). This means that CD163 activated with PD1 antibody should release sCD163, suggesting its utility as a prognostic marker for anti-PD1 antibody treatment. Indeed, serum levels of sCD163 were significantly increased in responders compared to non-responders 6 weeks after initial administration of nivolumab for cutaneous melanoma (84.6% sensitivity, 87.0% specificity; *p* = 0.0030) (24). Moreover, absolute serum levels of sCD163 after 6 weeks were significantly increased in patients treated with nivolumab who developed irAEs (*p* = 0.0018) (49). Those reports suggested that serum sCD163 could offer a predictive marker for the efficacy and irAEs of anti-PD1 antibodies.

In addition to sCD163, TAM-related chemokines could provide another group of prognostic markers for the outcomes of anti-PD1 antibody treatment (50). For example, CXCL5 is a chemokine that can recruit neutrophils, CXCR2+ myeloid-derived suppressor cells (MDSCs) and CXCR2+ monocytes. As we previously reported, production of CXCL5 from TAMs is increased by stimulation with periostin (51), which is detected in the stroma of cutaneous melanomas (23, 49). As we previously reported, baseline serum CXCL5 is associated with the efficacy of nivolumab in advanced melanoma (50) and increased serum levels of CXCL5 correlated significantly with irAEs from nivolumab (49). Unlike CXCL5, baseline serum concentrations of CXCL10 and CCL22 have not shown any correlations with the efficacy of nivolumab against advanced melanoma (50). These data suggested that TAM-related chemokines could further improve the predictive value of sCD163 systems in the future.

Although combination therapy with nivolumab and ipilimumab is recommended by the NCCN guideline for cutaneous melanoma as a first-line therapy (52), as described above, this combination therapy leads to a high frequency of SAEs among patients with advanced melanoma (9, 11). In the future, the evaluation of serum sCD163 as well as several TAM-related chemokines will undoubtedly play an important role in avoiding the administration of ipilimumab for patients who respond to anti-PD1 antibodies.

## Concluding Remarks

Although several studies have suggested useful predictive markers for the efficacy and irAEs of ICIs, exact methods to determine predictive markers remain under investigation. Further studies are needed to improve the systems for predicting the efficacy of ICI treatment.

## Author Contributions

YK, TF, and TH wrote manuscript. SA supervise the manuscript.

### Conflict of Interest Statement

The authors declare that the research was conducted in the absence of any commercial or financial relationships that could be construed as a potential conflict of interest.
